# Surgical Approach to Primary Hyperparathyroidism in Patients with Concomitant Thyroid Diseases: A Retrospective Single Center Study

**DOI:** 10.1155/2020/2182539

**Published:** 2020-02-22

**Authors:** Elena Castellano, Paolo Benso, Roberto Attanasio, Alberto Boriano, Corrado Lauro, Giorgio Borretta, Felice Borghi

**Affiliations:** ^1^Department of Endocrinology, Diabetes and Metabolism, Santa Croce and Carle Hospital, Cuneo, Italy; ^2^Unit of General and Oncological Surgery, Department of Surgery, Santa Croce and Carle Hospital, Cuneo, Italy; ^3^IRCCS Orthopedic Institute Galeazzi, Endocrinology Service, Milan, Italy; ^4^Medical Physics Department, Santa Croce and Carle Hospital, Cuneo, Italy

## Abstract

**Background:**

Primary hyperparathyroidism (PHPT) and thyroid diseases are a frequent concomitant occurrence, but the surgical approach to associated disease is still debated.

**Methods:**

We retrospectively evaluated a series of PHPT patients focusing on thyroid disease and surgery.

**Results:**

Among 238 PHPT patients undergoing parathyroidectomy (PTX) between 2002 and 2017, 128 were affected also by a benign thyroid disease, namely, goiter in 118 (76 multinodular (MNG) and 42 uninodular (UNG)), autoimmune thyroiditis in 10, and hyperthyroidism in 21. Surgical approach was unilateral neck exploration (UNE) in 59 patients and bilateral neck exploration (BNE) in 69. The PHPT cure rate was 94%. On comparing patients submitted to PTX only and PTX plus thyroidectomy (TX), in the latter MNG and hyperthyroidism were more frequent, and surgical time and length of stay were longer. No difference in surgical complications was found between patients undergoing UNE and BNE.

**Conclusion:**

PHPT patients with a concomitant thyroid disease underwent double surgery in almost two-thirds of the cases, mostly by BNE. The main factors driving the decision to perform concomitant PTX and TX were the presence of thyroid nodular disease with the nodule site ipsilateral to the presurgically localized parathyroid adenoma.

## 1. Introduction

Primary hyperparathyroidism (PHPT) and thyroid diseases are common in the general population (1–3), and a higher probability of identifying thyroid diseases in PHPT patients is probably due to the anatomic proximity of thyroid and parathyroid glands. The reported prevalence of the concomitant occurrence of these two clinical conditions is widely scattered (17–84%) (4–9), and we reported recently that the majority of PHPT patients resident in an endemic goiter area had a thyroid disease (10).

Despite the reported high prevalence of this association, the proper surgical approach when PHPT and thyroid diseases coexist is still a matter of debate. Recently, the American Association of Endocrine Surgeons (AAES) guidelines (11) recommended that thyroidectomy (TX) should be performed concomitantly with parathyroidectomy (PTX) whenever PHPT patients meet evidence-based surgical criteria for isolated thyroid disease.

We evaluated retrospectively a series of patients with PHPT looking for (1) the surgical approach performed in those with associated thyroid diseases and (2) the factors driving the decision to perform both PTX and TX.

## 2. Methods

### 2.1. Design

We retrospectively evaluated a surgical monocentric series of 238 patients with PHPT, undergoing PTX at our hospital between 2002 and 2017, focusing on concomitant thyroid disease, not suspicious for malignancy. In the selected period, the same team was in charge for neck surgery.

The study was conducted in accordance with the Declaration of Helsinki and was approved by the Institutional Review Board and the Ethical Committee of our institution. No informed consent was required for this study because we only retrospectively accessed a de-identified database for analysis purposes. All data were collected as part of routine clinical and psychological procedures.

### 2.2. Patients

Patients had been referred by general practitioners, primary care clinics, and subspecialty clinics. The diagnosis of PHPT had been established by the presence of hypercalcemia and concomitant inappropriately raised serum parathyroid hormone (PTH) levels on at least two separate occasions (reference range for calcium levels 8.4–10.2 mg/dL, for PTH vide infra). Patients diagnosed with multiple endocrine neoplasm, hyperparathyroidism-jaw tumor syndrome, or familial hypocalciuric hypercalcemia were excluded, as well as those with thyroid nodules cytologically suspicious for malignancy (Tir 4 and Tir 5, according to ATA guidelines—12).

No patient had been treated with drugs known to interfere with thyroid function (interferon, lithium, amiodarone, or immune checkpoint inhibitors).

All patients underwent imaging by neck ultrasonography (US) and ^99m^Tc-MIBI scintigraphy. Presurgical PHPT localization was considered positive if a single adenoma was disclosed by at least one of the two studies.

The US thyroid pattern was considered abnormal if nodules or features of chronic lymphocytic thyroiditis were found, defined as nodular or autoimmune disease, respectively.

According to history, ongoing therapy, and US results, patients were diagnosed with unilateral or bilateral goiter, hyperthyroidism, or chronic lymphocytic thyroiditis.

Depending on the extent of surgery, patients were grouped as follows: group 1, patients who underwent only PTX; group 2, patients who underwent both PTX and TX. Groups 1 and 2 were compared for demographic characteristics, PHPT biochemical markers, thyroid diseases, surgical approach, and length of hospital stay.

### 2.3. Methods

Calcium levels were assayed by automated analysis using colorimetric and enzymatic methods, while ionized serum calcium was analyzed by a specific probe after correction for pH.

Serum intact PTH concentrations were measured up to 2012 using a 2-site immunochemiluminometric assay (Immulite 2000; DPC, Los Angeles, CA) with inter- and intra-assay variation coefficients of 6.3 to 8.8% and 4.2 to 5.7%, respectively. Thereafter, serum intact PTH concentrations were measured using a new second-generation immunochemiluminometric assay (COBAS e411; ROCHE Diagnostics, Risch-Rotkreuz, Switzerland) with inter- and intra-assay variation coefficients of 3.1 to 6.5% and 1.4 to 3.2%, respectively. The corresponding normal ranges are 20 to 65 ng/L and 15 to 65 ng/L.

An intraoperative PTH (ioPTH) fall ≥50% at ten minutes vs. baseline was considered adequate for PHPT cure.

After primary surgery, PHPT was considered persistent if hypercalcemia recurred within 6 months or recurrent if hypercalcemia relapsed after at least 6 months of normocalcemia.

### 2.4. Statistical Analysis

Variables were preliminarily tested for normal distribution with the Shapiro–Wilks W-test, and data were expressed as mean ± SD when normally distributed as median and interquartile range (IQR) when not normally distributed.

Continuous variables with nonnormal and normal distribution were analyzed by Mann–Whitney *U* test and *t*-test for unpaired samples, respectively, as appropriate. Differences in categorical variables were analyzed by χ^2^ or Fisher's test as appropriate.

Logistic regression analysis was used to evaluate the correlations between the surgical approach and the other variables. Variables that were significant in univariate analysis were entered into multiple regression analysis.

The level of statistical significance was set at *p* < 0.05. The calculations were performed using SPSS (IBM SPSS Statistics, Version 21).

## 3. Results

Among the 238 PHPT patients undergoing PTX at our hospital in the considered period, 128 (53.8%) fulfilled the inclusion criteria.

Table 1 summarizes the characteristics of the whole series. The presurgical imaging resulted positive for a single adenoma in 101 patients (78.9%). Multinodular goiter (MNG), uninodular goiter (UNG), autoimmune thyroiditis (AIT), and hyperthyroidism were diagnosed in 76 (59.4%), 42 (32.8%), 10 (7.8%), and 21 (16.4%) patients, respectively. All 21 patients with hyperthyroidism had toxic MNG.

When thyroid nodules were ipsilateral to the parathyroid adenoma, PTX + TX was performed in the majority of patients (71%), and in 81% of them the surgical approach was unilateral; on the other hand, when thyroid nodules were contralateral, the combined surgery was performed only for 46.8% of patients, all of them with bilateral surgical approach. The mean diameter of thyroid nodules contralateral to parathyroid adenoma was 20 mm. Local compressive symptoms and hyperthyroidism were the most frequent causes driving concomitant PTX and TX.

Eighty percent of patients bearing bilateral thyroid nodules underwent concomitant PTX and TX, with BNE in the large majority of the cases (90%).

Surgical approach was thus unilateral neck exploration (UNE) in 59 (46.1%) patients and bilateral neck exploration (BNE) in 69 (53.9%). The PHPT cure rate was 94%.

Patients undergoing bilateral surgery had thyroid nodules contralateral to parathyroid adenoma (15/38) or bilateral nodular goiter (18/38) in the large majority of cases (33/38, 87%).

The comparison between group 1 and group 2 disclosed no differences for age, sex distribution, PHPT characteristics, and presurgical localization rate. In group 2 MNG and hyperthyroidism were significantly more frequent, and surgical time and length of stay were significantly longer (*p* < 0.0001).

In group 2, seven patients (8.3%) had an incidental identification of a thyroid cancer. It was a papillary microcarcinoma in 6 patients (mean diameter 5 mm; range 2–8 mm) and a 24 mm follicular carcinoma, associated to a 2 mm papillary microcarcinoma in the last patient. In none of them there was a preoperative suspicion of cancer.

Table 2 shows the comparison between patients undergoing surgery by UNE or BNE among group 2 patients. No differences were found in the demographic characteristics of patients. The type of thyroid disease significantly differs between the two subgroups, being UNG and AIT more frequent in patients submitted to UNE and MNG more frequent in patients undergoing BNE (*p* < 0.0001). The procedure length and the length of stay were significantly higher in patients submitted to BNE (*p* < 0.0001 and =0.011, respectively), while no difference in the complication rate was found between the two subgroups.

In univariate analysis, double surgery resulted significantly related to the type of thyroid disease, to the presence of hyperthyroidism, and to nodule site (ipsilateral or contralateral to the parathyroid adenoma). As reported in Table 3, in multiple analysis, only the type of thyroid disease and thyroid nodule site maintained statistical significance (*p*=0.05 and =0.044, respectively).

In univariate analysis, the unilateral approach resulted significantly correlated to the type of thyroid disease, to the presence of hyperthyroidism, and to the positive presurgical PHPT localization. In multiple analysis (Table 4), only the presurgical PHPT localization maintained statistical significance (*p* < 0.0001).

## 4. Discussion

Synchronous thyroid pathology has been reported to occur in 17 to 84% of patients with PHPT [1–5]. More than half of surgically treated PHPT patients of our cohort had a concomitant thyroid disease.

About two-thirds of our PHPT patients with concomitant thyroid disease without any suspicion of malignancy underwent simultaneous thyroid and parathyroid surgery. Patients undergoing combined surgery were submitted to BNE more frequently than patients undergoing PTX alone, which in turn were submitted to unilateral approach in most cases. The combined surgery was associated to the type of thyroid disease and to the contralateral nodule site relative to parathyroid adenoma, while UNE was associated to the positive presurgical localization of parathyroid adenoma.

The combined thyroid and parathyroid surgery had similar cure rate and complication rate despite longer procedure length and hospital stay than PTX alone.

PHPT is very frequently associated with thyroid abnormalities [6–8] and, on the other hand, the reported prevalence of PHPT in patients bearing any thyroid disease is three times higher than in healthy subjects [7]. In an unselected series in an endemic goiter area, we recently reported that 60% of our PHPT patients had also a thyroid disease, which was unknown prior to PHPT diagnosis in almost one-third of cases [10].

The association between PHPT and thyroid disease still remains to be fully understood. Some authors suggested that it may be coincidental due to close surveillance of the thyroid gland during PTX, while others postulated a goitrogen role of the long-term exposure to elevated calcium levels [9, 11].

Regardless of any causal association, the presence of a thyroid disease plays a relevant role in the clinical management of PHPT patients. Actually, a lower sensitivity and specificity of preoperative parathyroid adenoma localization in patients with coexisting thyroid disease has been extensively reported [5, 9, 12, 13]. In addition, hyperthyroidism could worsen some PHPT features, such as hypercalcemia and osteoporosis [10]. Moreover, the prevalence of thyroid cancer seems to be higher in patients with PHPT, with thyroid malignancy reported in 2 to 12% of PHPT surgical patients [13, 14].

Finally, the surgical approach to PHPT may be changed if thyroid disease is also present [9, 15, 16]. While the simultaneous TX and PTX are mandatory in case of malignancy suspicion, the surgical approach in case of concomitant thyroid disease unsuspected for malignancy is still debated [9, 15, 16]. Italian AME guidelines [17] in 2012 suggested to treat nodular thyroid disease concomitantly to PHPT, in order to avoid repetitive surgery that would be more worrisome owing to postsurgical adhesions. More recently, AAES [18] recommended that TX should be performed concomitantly with PTX whenever PHPT patients meet evidence-based surgical criteria for isolated thyroid disease.

Few surgical series [12, 15, 19] of PHPT patients with concomitant thyroid disease reported an extensive use of simultaneous surgery, with BNE more frequently performed in comparison to patients without thyroid disease. Although the majority of concurrent thyroid disease resulted was benign, a not negligible prevalence of incidentally discovered thyroid malignancy was previously reported. In particular, papillary microcarcinoma was disclosed in 12% of the series of 103 patients reported by Bentrem et al. [12], 17.6% among the 51 patients reported by Kösem et al. [19], 13% among the 85 patients reported by Latina et al. [10], and 32.2% of the 231 patients belonging to a series by Scerrino et al. [15].

In agreement with previous data, in our retrospective study, simultaneous surgery was performed in the majority of cases, mostly with the bilateral approach. This decision has a number of possible explanations. First, some PHPT patients with benign nodular goiter have undergone thyroid surgery for hyperthyroidism or symptoms of tracheal compression. Nodular goiter complications are a common occurrence in endemic goiter areas [20], so that a simultaneous surgery could avoid costs and risks associated with neck reexploration. Finally, this approach can detect unsuspected occult thyroid cancers that are more prevalent in PHPT patients than in general population [13, 14].

In conclusion, this retrospective study showed that PHPT patients with a concomitant thyroid disease underwent simultaneous parathyroid and thyroid excision in almost two-thirds of the cases. In these patients, the BNE is performed more frequently than in patients undergoing PTX alone, with similar cure rate and incidence of permanent surgical complication. The main factors driving the decision to perform associated PTX and TX are thyroid nodular disease and nodule site ipsilateral to presurgically localized parathyroid adenoma.

## Figures and Tables

**Table 1:** Demographic, biochemical, and clinical characteristics of patients with PHPT and concomitant thyroid disease with the comparison between patients undergoing only PTX (group 1) or PTX + TX (group 2).

	Whole series (*n* = 128)	Group 1 (*n* = 44)	Group 2 (*n* = 84)	*p* ^*∗*^
Age (years)	64.1 ± 9.8	63.1 ± 9.5	64.6 ± 9.9	0.411

Males	28 (21.9%)	9 (20.5%)	19 (22.6%)	0.955

PHPT clinics:				0.597
Symptomatic	73 (57%)	27 (61.4%)	46 (54.8%)	
Asymptomatic meeting surgical criteria	55 (43%)	17 (38.6%)	38 (45.2%)	

Serum total calcium (mg/dL)	11.1 ± 1.2	11.4 ± 1.6	11 ± 0.9	0.071

PTH (ng/L)	144 [115]	136 [92.5]	144 [118.9]	0.96

Thyroid disease:				**<0.0001**
MNG	76 (59.4%)	16 (36.4%)	60 (71.4%)	
UNG	42 (32.8%)	20 (45.4%)	22 (26.2%)	
AIT without nodules	10 (7.8%)	8 (18.2%)	2 (2.4%)	

Hyperthyroidism	21 (16.4%)	2 (4.5%)	19 (22.6%)	**0.018**

Presurgical localization:				0.722
Positive for a single adenoma	101 (78.9%)	36 (81.8%)	65 (77.4%)	
Negative or suspicious for multiglandular disease	27 (21.1%)	8 (18.2%)	19 (22.6%)	

Surgery:			
UNE	59 (46.1%)	36 (81.8%)	23 (27.4%)	**<0.0001**
BNE	69 (53.9%)	8 (18.2%)	61 (72.6%)	
ioPTH fall	85.9%	84.1%	86.9%	0.867

Persistent PHPT	6 (4.7%)	2 (4.5%)	4 (4.8%)	0.700

Recurrent PHPT	2 (1.6%)	1 (2.3%)	1 (1.2%)	0.778
Postsurgical hypocalcemia:			
Transient	13 (10.2%)	3 (6.8%)	10 (11.9%)
Persistent	1 (0.8%)	0	1 (1.2%)

Surgical site infection	3 (2.3%)	1 (2.3%)	2 (2.4%)	0.172°

RLN monolateral palsy:			
Transient	3 (2.3%)	0	3 (3.6%)
Persistent	1 (0.8%)	0	1 (1.2%)

Procedure length (minutes)	104 ± 47 [15–290]	79 ± 34 [15–190]	119 ± 48 [35–290]	**<0.0001**

Hospital stay (days)	2.4 ± 1.5 [0–9]	1.6 ± 1 [0–5]	2.8 ± 1.5 [1–9]	**<0.0001**

Abbreviations: AIT = autoimmune thyroiditis; BNE = bilateral neck exploration; MNG = multinodular goiter; ioPTH = intraoperative PTH; RLN = recurrent laryngeal nerve; UNG = uninodular goiter; UNE = unilateral neck exploration. Data are expressed as mean ± SD when normally distributed, median and (interquartile range) when not normally distributed, and as percentage and absolute number when categorical. ^*∗*^The comparison between group A and group B. °Refers to the comparison of the whole incidence of surgical complications.

**Table 2:** Comparison between UNE and BNE in patients of group 2.

	UNE (*n* = 23)	BNE (*n* = 61)	*p* ^*∗*^
Age (years)	63.9 ± 10.5	64.9 ± 9.8	0.681
Males	7 (30.4%)	12 (19.7%)	0.448

Thyroid disease:			**<0.0001**
MNG	11 (47.8%)	49 (80.3%)	
UNG	10 (43.5%)	12 (19.7%)	
AIT without nodules	2 (8.7%)	0	

Hyperthyroidism	3 (13%)	16 (26.2%)	0.319
Tir3 at FNAB	2 (8.7%)	5 (8.2%)	0.787

Presurgical localization:			0.114
Positive for a single adenoma	21 (91.3%)	44 (72.1%)	
Negative or suspicious for multiglandular disease	2 (8.7%)	17 (27.9%)	

Partial TX	23 (100%)	27 (44.3%)	**<0.0001**

ioPTH fall >50% at 10 min	22 (95.7%)	51 (83.6%)	0.272
Persistent PHPT	0	4 (6.6%)	—
Recurrent PHPT	0	1 (1.6%)	

Postsurgical hypocalcemia:			0.418°
Transient	2 (8.7%)	8 (13.1%)	
Persistent	0	1 (1.6%)	
Surgical site infection	0	2 (3.3%)	

RLN monolateral palsy:		
Transient	0	3 (4.9%)
Persistent	0	1 (1.6%)
Procedure length (minutes)	84 ± 23 [35–150]	130 ± 50 [19–290]	**0.0001**

Hospital stay (days)	2.2 ± 0.7 [1–4]	3.1 ± 1.6 [1–9]	**0.011**

Abbreviations: AIT = autoimmune thyroiditis; BNE = bilateral neck exploration; FNAB = fine needle aspiration biopsy; MNG = multinodular goiter; ioPTH = intraoperative PTH; RLN = recurrent laryngeal nerve; TX = thyroidectomy; UNG = uninodular goiter; UNE = unilateral neck exploration. Data are expressed as mean ± SD when normally distributed, median and interquartile range when not normally distributed, and as percentage and absolute number when categorical. ^*∗*^The comparison between group A and group B. °Refers to the comparison of the whole incidence of surgical complications.

**Table 3:** Multiple logistic regression analysis for TX.

	*p*	ODDS	95% CI
Type of thyroid disease	**0.05**	**0.38**	**0.14–1.00**
MNG			
UNG			
AIT			

Hyperthyroidism (yes)	0.418	2.66	0.49–14.42

Thyroid nodule site:	**0.044**	**0.49**	**0.25–0.95**
Bilateral			
Ipsilateral			
Contralateral			

Abbreviations: AIT = autoimmune thyroiditis; MNG = multinodular goiter; TX = thyroidectomy; UNG = uninodular goiter; UNE = unilateral neck exploration.

**Table 4:** Multiple logistic regression analysis for BNE.

	*p*	ODDS	95% CI
Type of thyroid disease	0.09	0.38	0.11–1.02
MNG			
UNG			
AIT			

Hyperthyroidism (yes)	0.418	1.66	0.49–5.66

Parathyroid presurgical localization	**0.001**	**0.14**	**0.04–0.45**
Positive for single adenoma			
Negative or multiglandular			

Thyroid nodule site	0.537	0.84	0.48–1.47
Bilateral			
Ipsilateral			
Contralateral			

Abbreviations: AIT = autoimmune thyroiditis; BNE = bilateral neck exploration; MNG = multinodular goiter; UNG = uninodular goiter.

## Data Availability

The datasets generated during and/or analyzed during the current study are not publicly available but are available from the corresponding author upon reasonable request.

## Abbreviations

AAES: American Association of Endocrine Surgeons
AIT: Autoimmune thyroiditis
ATA: American thyroid association
BNE: Bilateral neck exploration
FNAB: Fine needle aspiration biopsy
ioPTH: Intraoperative PTH
IQR: Interquartile range
MIBI: Methoxyisobutylisonitrile
MNG: Multinodular goiter
PHPT: Primary hyperparathyroidism
PTH: Parathyroid hormone
PTX: Parathyroidectomy
RLN: Recurrent laryngeal nerve
TX: Thyroidectomy
UNE: Unilateral neck exploration
UNG: Uninodular goiter
US: Ultrasonography.
